# Introducing the Cancer Microenvironment Section of Journal of Translational Medicine

**DOI:** 10.1186/1479-5876-8-60

**Published:** 2010-06-22

**Authors:** Fernando Vidal-Vanaclocha, Isaac P Witz

**Affiliations:** 1 Institute of Applied Molecular Medicine (IMMA) and University Hospital of Madrid Scientific Foundation, CEU-San Pablo University School of Medicine, Boadilla del Monte, 28668 Madrid, Spain; 2 Department of Cell Research & Immunology, Faculty of Life Sciences, Tel Aviv University, Ramat Aviv, Tel Aviv, 69978, Israel

## Editorial

BioMed Central is proud and delighted to announce the new section of the *Journal of Translational Medicine *devoted to promoting the field of Cancer Microenvironment.

### A new forum on diagnostic and therapeutic implications of molecular biomarkers and targets from the cancer microenvironment

The "Seed and Soil" theory, proposed by Stephen Paget over 120 years ago (1889) [1], laid the foundations for the modern concept of the tumor microenvironment (TME) and its involvement in organ-specific metastasis. In contemporary clinical oncology TME research is focused on the elucidation of tumor-microenvironment interactions, at different clinical phases, that contribute to cancer progression and metastasis. The understanding of the constellation of cancer cells with various factors in the microenvironment is an essential prerequisite for rational therapeutically targeting of metastasis-promoting tumor-microenvironment interactions (for review, see Witz, 2008) [2].

The field of TME research is propagating steadily. Recent discoveries in this area include: mechanisms of cancer cell regulation at metastatic niches; identification of functional links between biologic background at specific organs and incidence and progression of cancer and metastasis; delineating tumorigenic and pro-metastatic effects of infectious agents, insight into the effects of inflammatory factors, hypoxia and oxidative stress on cancer progression; findings related to the origin and unique features of cancer-associated fibroblasts, macrophages and bone marrow-derived cells; tissue architecture and extracellular matrix associated to tumor stromagenesis and angiogenesis; cancer cell dormancy; and community effects among heterogeneous cancer cell subpopulations, including cancer stem cells.

Translational cancer research is a well consolidated activity at the academic, industrial and governmental levels. In contrast, there is a lot to be done with respect to translating the insights gained in the laboratory on the involvement of the TME in cancer pathogenesis to the clinic and the biomedical industry. Translational research that bridges the laboratory and the clinic is still under development, and this new section of the JTM is especially interested in the publication of discoveries in four areas:

1) Clinical models that allow the characterization of the molecular profile of non-cancer cells in the TME and of their soluble products, and how these cells can be regulated at different stages of cancer progression.

2) Molecular mechanisms that govern the establishment of subclinical metastatic niches, and biomarkers that characterize the cellular components of such niches. Such studies might lead to novel approaches to detect, prevent or cure metastasis.

3) Studies seeking to correlate defined cancer microenvironment biomarkers with incidence, response to and outcome of therapy.

4) Laboratory studies of new drugs and biological agents that can target the TME and exert beneficial influences on cancer patients.

The focus of this new section of the JTM is on studies that bridge the laboratory and the clinic. We intend to publish original articles describing experimental or clinical data on the cellular and molecular components of the TME with implications for prevention, diagnosis, and therapy of human cancers.

There are boundless possibilities offered by a rapidly published, open-access, online communication of results and exchange of opinions. The Cancer Microenvironment section of JTM is a potent tool to most efficiently achieve these tasks. Thus, this section of the *JTM *provides a venue for publication of original research articles, literature reviews, opinion/position papers, and a forum to discuss the hot issues in the cancer microenvironment. Therefore, we have the honor and pleasure to invite you to submit your work, your thoughts and/or to engage in informal discussions. We welcome you and look forward to your contributions.
